# Recent advances in therapeutic use of transforming growth factor-beta inhibitors in cancer and fibrosis

**DOI:** 10.3389/fonc.2025.1489701

**Published:** 2025-04-25

**Authors:** Hanhui Jing, Yan Gao, Linyuan Jing, Hanyu Yang, Shanglong Liu

**Affiliations:** ^1^ Department of Gastrointestinal Surgery, the Affiliated Hospital of Qingdao University, Qingdao, Shandong, China; ^2^ Department of Integrated Chinese and Western Medicine, Yantai Yuhuangding Hospital, Yantai, Shandong, China

**Keywords:** transforming growth factor-beta, cancer-associated fibrosis, targeted therapies, cancer biology, epithelial-mesenchymal transition

## Abstract

Transforming growth factor-beta (TGF-β) has long been known to be associated with early embryonic development and organogenesis, immune supervision, and tissue repair and homeostasis in adults. TGF-β has complex roles in fibrosis and cancer that may be opposing at different stages of these diseases. Under pathological conditions, overexpression of TGF-β causes epithelial–mesenchymal transition, deposition of extracellular matrix, and formation of cancer-associated fibroblasts, leading to fibrotic disease or cancer. Fibroblasts, epithelial cells, and immune cells are the most common targets of TGF-β, while fibrosis and cancer are the most common TGF-β-associated diseases. Given the critical role of TGF-β and its downstream molecules in fibrosis and progression of cancer, therapies targeting TGF-β signaling appear to be a promising strategy. Preclinical and clinical studies have investigated therapies targeting TGF-β, including antisense oligonucleotides, monoclonal antibodies, and ligand traps. However, development of targeted TGF-β therapy has been hindered by systemic cytotoxicity. This review discusses the molecular mechanisms of TGF-β signaling and highlights targeted TGF-β therapy for cancer and fibrosis as a therapeutic strategy for related diseases.

## 1 Background

Transforming growth factor-beta (TGF-β) is a multifunctional polypeptide cytokine that belongs to the TGF-β superfamily and plays an important role in multiple biological processes, including cell growth, differentiation, apoptosis, the immune response, and wound healing (1, 2). Accurate TGF-β signaling is essential for normal function and homeostasis in humans, and abnormalities of TGF-β can lead to a variety of diseases (2). However, TGF-β has dual functionality. In normal cells, TGF-β halts the cell cycle in G1 phase and can inhibit cell proliferation and promote apoptosis (3). In contrast, cancer cells become resistant to inhibition by TGF-β signaling through mutations or epigenetic modifications. In the tumor microenvironment (TME), TGF-β contributes to tumor growth, invasion, and spread, and advanced tumors produce an excessive amount of TGF-β that promotes proliferation of tumor cells, vascular cells, immune cells, and fibroblasts (4–7). These processes convert TGF-β into a tumor-promoting agonist as the disease progresses. TGF-β functions as a tumor promoter by stimulating tumor cells to undergo what is known as epithelial-mesenchymal transition (EMT). EMT can activate angiogenesis and cancer-related fibroblasts and enable tumors to evade inhibitory immune responses, further promoting growth and progression of cancer (8).

**Figure 1:**
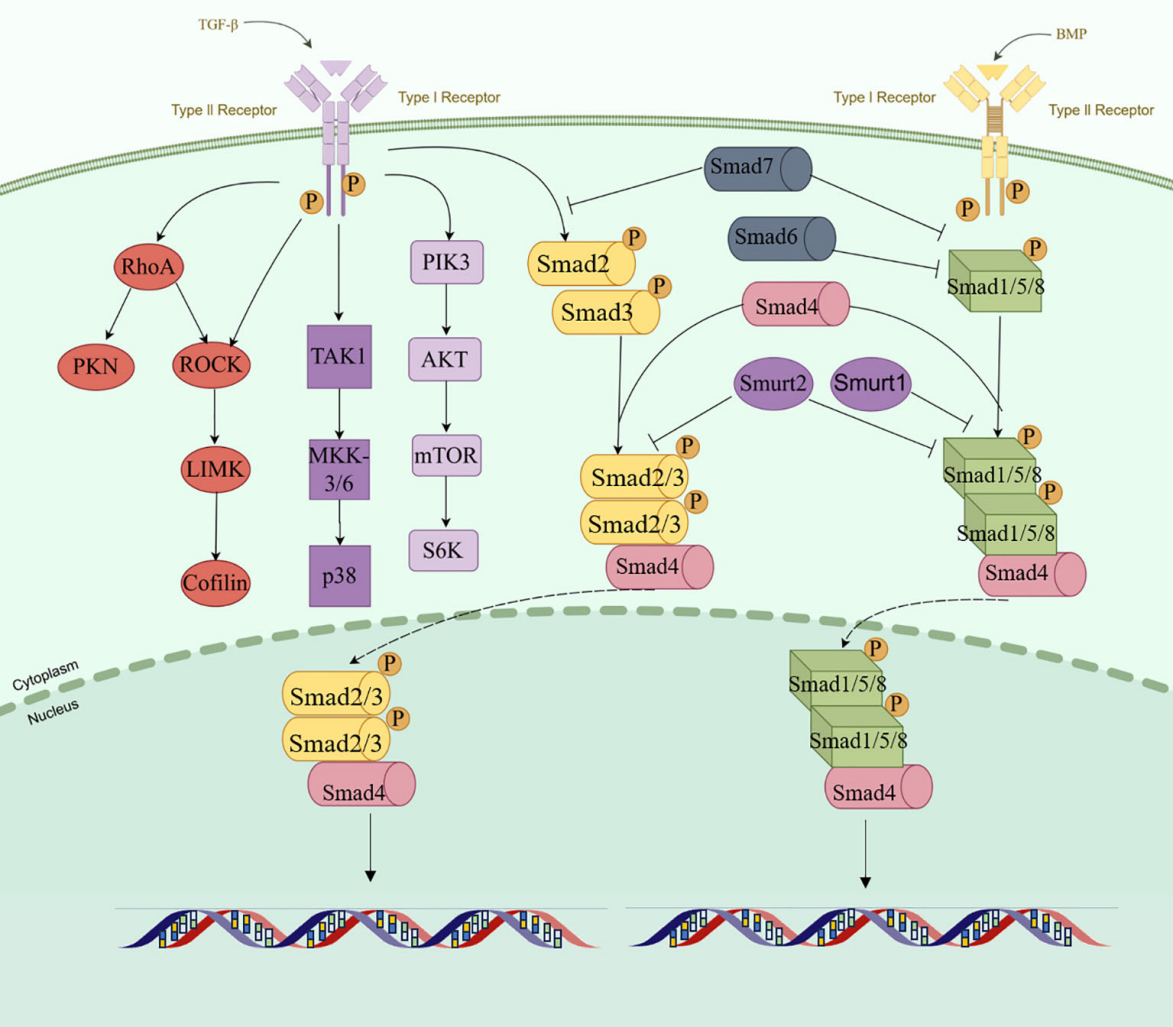
Schematic representation of the TGF-β activation pathway. (By Figdraw.).

In recent years, researchers have found that inactivating the TGF-β signaling pathway in CD4+ cells leads to increased production of interleukin-4 by T helper 2 cells in mouse models of breast cancer and regression of the disease (9, 10). Given the important role of TGF-β in human fibrosis and cancer, anti-TGF-β approaches have been used to treat several diseases, including melanoma, colorectal cancer, and breast cancer (11–13). A growing body of preclinical and clinical data suggests that blocking TGF-β signaling is an effective treatment for cancer and fibrosis. This review focuses on the latest advances in use of TGF-β inhibitors for therapeutic management of these diseases. (see **Figure 1**).

## 2 TGF-β signaling in cancer and fibrosis

TGF-β is a multifunctional polypeptide cytokine and has three mammalian subtypes, namely, TGF-β1, TGF-β2, and TGF-β3, which are encoded by different genes but act through the same signaling system (14–16). During the synthesis of TGF-β in cell, TGF-β precursors are hydrolyzed by the endopeptidase furin in the Golgi apparatus and then transported to and bound to the extracellular matrix (ECM) together with the latency-associated peptide (LAP) in the amino terminal region (17–19). In the extracellular space, the LAP/TGF-β complex can be cleaved by a variety of proteases to release active TGF-β, including plasmin, matrix metalloproteinase (MMP) 2, and MMP9 (20, 21).TGF-β influences multiple processes, including cell growth and differentiation, apoptosis, cell motility, angiogenesis, and the immune response (22–25). These effects are heavily dependent on the TME, including tumor hypoxia. This pleiotropy is manifested in the following process: TGF-β is released from ECM. Subsequently, it binds to the transmembrane TGF-β receptor type II (TβRII) on the target cell and recruits the type I receptor (TβRI) to exert its function. The phosphorylation of type I receptor by type II receptor and subsequent phosphorylation of R-SMAD is a central feature of the TGF-β family of ligand signaling mechanisms (26, 27). TβRII is transphosphorylated, activated, and signals through phosphorylation of the SMAD 2/3 protein. In the process of signal transmission, auxiliary receptors facilitate or inhibit signal transmission through TGF-β receptors. Among them, endoglin can promote signal transmission to regulate angiogenesis, while BAMBI interferes with the signaling of receptor complexes, leading to the association of BAMBI expression with various human diseases, including cancer and tissue fibrosis (28–31). The key regulatory proteins in the TGF-β intracellular signaling axis mainly include the SMAD protein family, such as SMAD2 and SMAD3, which are receptor-regulated SMADs. Phosphorylation allows SMAD 2/3 to form heteromeric complexes with SMAD 4 proteins and translocate into the nucleus. By binding to transcription factors, SMAD complexes affect mRNA splicing, stability, and translation through RNA-binding proteins (RBPs) and non-coding RNAs (ncRNAs) (32, 33). SMAD can also control the maturation process of miRNAs. In addition, TGF-β/SMAD signaling can modulate the expression of several long non-coding RNAs (lncRNAs) that act as mediators of TGF-β reactions (34). R-Smads and Smad4 are not highly regulated by extracellular signaling in most cell types (35). Ubiquitin-proteasome-mediated degradation controls the levels of post-translational Smads, and E3 ubiquitin ligase, Smurf1, and Smurf2 antagonize TGF-β conduction by interacting with and targeting R-Smad (36). Following TGF-β signaling, phosphorylated Smad2 or Smad3 can form stable complexes with Smurf2. Thus, the majority of nucleus Smad2 or Smad3 is not a target for degradation, but is dephosphorylated and relocalized into the cytoplasm (37–39). In contrast to R-Smads, Smad4 is not normally subject to ubiquitin-mediated degradation, and some tumor-associated mutations allow ubiquitination or reduce the stability of Smad4 (40, 41). The initiation and propagation of TGF-β signaling are counteracted by the activities of SMAD6 and SMAD7, which are inhibitory SMADs (I-SMAD) (42). SMAD7 antagonizes the TGF-β pathway by recruiting the E3 ubiquitin ligases SMURF1 and SMURF2 to the type I receptor, promoting its ubiquitination and subsequent degradation, making it a more effective inhibitor of TGF-β signaling than SMAD6, which preferentially inhibits BMP signaling (43, 44). Although there is a typical SMAD-dependent pathway, TGF-β also initiates SMAD-independent signal transduction. Research indicates that ERK activation occurs in cholesterol-rich lipid rafts in human keratinocytes. The regulation of JNK and p38 MAP kinase pathways is crucial in inflammation, cell differentiation, and apoptosis, where TGF-β-activated kinase 1 (TAK1) also serves as a classic inhibitory regulator of TGF-β signaling (45, 46). The MAPK and phosphoinositide 3-kinase pathways also have potent agonists. Similarly, stress sensors regulate the activation of p38 MAPK and c-Jun amino-terminal kinase, while tumor necrosis factor, interleukin-1, and Toll-like receptors control activation of TAK1. Therefore, the role of TGF-β in the regulation of these pathways under normal physiological conditions and in disease remains difficult to determine.

Dysregulation of TGF-β signaling is associated with many pathological processes, including tumor progression and fibrosis (47). In advanced malignant disease, tumor cells undergo genetic and/or epigenetic changes that gradually render them insensitive to TGF-β by weakening the pathway via which TGF-β inhibits their growth and accumulate mutations in the TGF-β signaling cascade that allow them to evade the antitumor surveillance activity of TGF-β. Examples of such escape include SMAD4 mutations in gastric cancer and TβRI mutations in colon cancer, where loss of the functional mutant component of TGF-β is not sufficient to cause tumorigenesis but promotes transformation of precancerous cells to a more malignant phenotype (48–51). In the later stages of tumor development, TGF - β, through the secreted TGF - β protein, plays a multifaceted role. It stimulates cell proliferation, triggers the formation of new blood vessels, and strengthens the immunosuppressive state of the tumor (52). The carcinogenic events that lead to TGF-β switching are diverse. For example, co-activation of KRAS, inactivation of SMAD4, and changes in the CDKN2A genome lead to rapid transformation of non-invasive pancreatic intraepithelial neoplasms into invasive pancreatic ductal adenocarcinomas, a large number of which are related to tumor stage, with the SMAD4 inactivation rate in high-grade tumors reaching up to 31% (53–56). TGF-β increases the ability of cells to migrate and invade neighboring tissues via EMT, allowing epithelial cells to change from a cuboidal shape to an elongated spindle shape and an invasive phenotype (57). During EMT, epithelial cells lose E-cadherin and zonula occludens-1 proteins from the plasma membrane and upregulate their expression of vimentin, fibronectin, and N-cadherin, thereby increasing their mobility (58, 59). Studies in cultured cells and mice have shown that remodeling of the ECM can release the underlying TGF-β complex and interact with inflammatory mediators, resulting in a stiffer ECM, and also increased metastasis of hepatocellular carcinoma in rats (60). Furthermore, the TGF-β-regulated immunosuppressive microenvironment indirectly promotes tumor escape, inhibits natural killer cells, and regulates the proliferation of macrophages, antigen-presenting dendritic cells, and granulocytes to control the development and function of the innate immune system (61–63). Deletion or mutations of SMAD4 in NK cells leads to impairment of NK cell homeostasis, and NK cell immune surveillance against metastases (64).

In addition to its effects on tumor development, TGF-β-induced EMT leads to fibrosis in the tissues of many organs, including the lungs and liver (65). Fibrosis involves necrosis of parenchymal cells and excessive deposition of ECM, which results in connective tissue hyperplasia, fibrosis, and eventually organ failure. Fibrosis also entails conversion of fibroblasts into cancer-associated fibroblasts (CAFs) (66). All three isoforms of TGF-β have fibrotic effects, and TGF-β is considered a major factor in both classical and non-classical signaling pathways in fibrosis, affecting the liver, kidney, and lung (67–69). Dysregulation of TGF-β leads to excessive deposition of ECM, which promotes pathological fibrosis and tumorigenesis and is also associated with migration of tumor cells. Furthermore, studies in zebrafish have shown that breast and prostate cancers promote tissue fibrosis and migration of cancer cells in the unique microenvironment provided by TGF-β produced in CAFs (70–72). Other studies have demonstrated a strong association between pulmonary fibrosis and an increased risk of lung cancer. This pro-fibrotic state stimulates increased expression of TGF-β, which is followed by crosstalk between or activation of downstream pathways, such as SMAD3 or other atypical pathways, leading to resistance of cancer cells to apoptosis and progression of cancer via CAFs (68, 73, 74). Abnormal accumulation of ECM triggers fibrotic and immunosuppressive processes by linking SMAD4, BRAF, and TP53 mutations with MYC amplification and produces a CAF phenotype (66, 75–78). In breast cancer, TGF-β not only promotes tumor growth but also forms a stable breast cancer stem cell phenotype by changing the metabolic reprogramming of CAFs. EMT of breast cancer cells is regulated by a TGF-β/SMAD-dependent pathway and activated by tumor necrosis factor-alpha/nuclear factor-kappa B/Twist, promoting metastasis (79, 80). Calon A et al. found that all poor prognostic features in colorectal cancer patients had elevated TGF-β expression, and in colon cancer, CAF produced TGF-β, which in turn activated the gene expression program in CAF. On the other hand, TGF-β in CAFs improves the tumor-initiating capacity of colorectal cancer cells, even in patients classified as having a good prognosis, and if CAF levels are elevated, this property increases metastatic potential and the ability to regenerate disease after treatment (81–84).

## 3 Strategies for inhibition of TGF-β

Given that the mechanisms of tissue fibrosis and tumorigenesis are inextricably linked and TGF-β signaling plays an important role in both conditions, inhibition of TGF-β is an area of intense interest in cancer research. Inhibitors of TGF-β signal transduction have been investigated in preclinical studies and are divided into five types according to whether their mechanism of action entails blocking the synthesis of TGF-β, blocking the TGF-β ligand, blocking activation of latent TGF-β, blocking the TGF-β receptor, or blocking intracellular signal transduction (85).

### 3.1 Blocking synthesis of TGF-β

Transcription and translation, two crucial steps in gene expression, are key to converting genetic information into the protein - based material essential for biological functions. One strategy is to use antisense oligonucleotides, which are complementary to mRNA targets and generate single-stranded deoxyribonucleotides. One such agent is trabedersen (AP-12009), an 18-polymeric thiophosphate-modified antisense oligodeoxynucleotide that is complementary to TGF-β2 mRNA (86). Developed by Antisense Pharma GmbH (Regensburg, Germany), trabedersen is specifically designed for clinical use in patients with highly aggressive TGF-β2-overexpressing tumors, such as malignant melanoma and high-grade glioma. In a cell line established from a patient with high-grade glioma, trabedersen led to a significant reduction in synthesis of TGF-β2 protein and inhibited cell proliferation and migration. Trabedersen was also well tolerated in a safety study by Schlingensiepen et al. in animals (87, 88). In a Phase I/II study, seven patients achieved stable disease and two achieved complete remission (88, 89). These encouraging results led to a Phase IIb clinical trial (NCT00431561) in which the efficacy and safety of trabedersen were assessed in patients. In this study, trabedersen was well tolerated with no serious adverse events and performed better than conventional chemotherapy in terms of median survival. At present, large Phase III trials of trabedersen are underway in patients with high-grade glioma. Phase I trials of this agent have also been initiated in patients with pancreatic cancer and colon cancer after it was found to inhibit the proliferation and migration of pancreatic cancer cells in mice (90, 91). ISTH0036 is an antisense oligonucleotide developed by Isarna Therapeutics GmbH (Munich, Germany) that targets TGF-β2 mRNA. Pfeiffer et al. evaluated the ability of this agent to inhibit fibrosis after glaucoma filtration surgery in a Phase I clinical trial and confirmed its safety and potential antifibrotic activity (92). However, there are challenges in the development of antisense oligonucleotides as a therapeutic target, including off-target effects, delivery to target tissues, and RNA-binding affinity.

### 3.2 Blocking the TGF-β ligand

Therapies that block the TGF-β pathway have been successfully developed for a variety of cancers. Neutralizing antibodies are the preferred approach because they bind directly to the ligand and block into the receptor. Thus far, three drug candidates have been investigated in clinical trials by Cambridge Antibody Technology (Cambridge, UK), namely, lerdelimumab (CAT-152), metelimumab (CAT-192) and fresolimumab (CAT-193) (93, 94). Blocking TGF-β ligands from binding to the receptor by using ligand traps or neutralizing antibodies is a promising strategy in cancer therapy. Among the neutralizing antibodies, fresolimumab (GC1008) is a human monoclonal antibody designed to neutralize all three subtypes of TGF-β and is the most widely investigated agent in this class (95). Soluble TGF-β receptors are another effective strategy for blocking binding of TGF-β ligands to cell receptors. The Phase I clinical study of AVID200 is currently in progress (96). Chen et al. discovered that in a mouse model of pancreatic ductal adenocarcinoma (PDAC), AVID200 is capable of regulating the heterogeneity of cancer - associated fibroblasts (CAFs) and significantly decreasing tumor metastasis to the liver (97).

#### 3.2.1 Studies in cancer

Fresolimumab has been investigated as a pan-TGF-β neutralizing antibody in patients with advanced melanoma and renal cell carcinoma, some of whom achieved stable disease or remission (98–100). A preclinical study in a mouse model of 4T1 breast cancer showed that use of 1D11 (Genzyme Corporation, Cambridge, MA, USA), another pan-TGF-β neutralizing antibody, slowed tumor growth, particularly when combined with radiotherapy. These findings indicated that the efficacy of TGF-β inhibitors depends on the combination of tumor parenchyma and microenvironment and drugs (101–103). Combination of fresolimumab with a programmed cell death protein 1 (PD-1) inhibitor has also shown promising results in a variety of cancers. However, although fresolimumab has a similar affinity for all TGF-β subtypes, these subtypes are expressed in varying amounts in different cancers, and thus antagonists need to be developed with specific targets. Accordingly, Sanofi Aventis has suspended clinical development of fresolimumab as an oncologic agent. Nevertheless, development of antibodies against fibrosis continues.

Similarly to neutralizing antibodies, soluble TGF-β receptors can interfere with or block the interaction between ligands and membrane binding receptors, acting as a “ligand trap.” Soluble TGF-β receptors have shown antitumor efficacy in preclinical mouse models of mesothelioma, liver cancer, and pancreatic cancer, with increased apoptosis of primary tumors and decreased metastasis (104, 105). TGF-β type III receptors bind to all subtypes of TGF-β. Furthermore, studies in the MDA-MB-23 breast cancer cell line showed that reduction of serum TGF-β levels decreased the movement and invasiveness of tumor cells and metastasis to the lungs (106–108).

#### 3.2.2 Studies in fibrosis

Many studies have shown that TGF-β1 signaling plays an important role in the pathogenesis of fibrosis. TGF-β is an effective promoter of fibrosis in cardiac fibrosis and idiopathic or interstitial pulmonary fibrosis (109). In various clinical trials, direct neutralization of TGF-β has been shown to have an anti-fibrotic effect. AVID200 is an engineered TGF - β ligand trap. It exhibits higher sensitivity to TGF - β1 than to TGF - β2, where TGF - β2 is a positive factor for hematopoiesis and cardiac function. As a result, AVID200 is more precisely targeted in treating anemia, such as that associated with myelodysplastic syndrome. Moreover, its ability to increase platelet count represents an effect not previously observed in other therapeutic approaches (8, 110). Systemic sclerosis is characterized by excessive deposition of ECM components in tissues and organs resulting in fibrosis. Metelimumab has been evaluated as an early treatment for systemic sclerosis but has not shown a therapeutic effect (111). In a Phase I trial (NCT01284322), biomarkers of systemic sclerosis (thrombospondin 1 and cartilage oligomeric protein) declined rapidly after treatment with fresolimumab. These findings suggest that fresolimumab holds promise as a treatment for systemic sclerosis. The US Food and Drug Administration has approved disitertide (P144), a peptide derived from the human beta-glycan ligand-binding domain, for testing in cutaneous fibrosis (112).

### 3.3 Blocking activation of latent TGF-β

Blocking the conversion of latent TGF-β to activated TGF-β has become an attractive target. Integrin αvβ6 is usually detected on fibrotic and remodeled cells and can promote invasion of cancer cells in several solid tumors (113). Overexpression of integrin αvβ6 is associated with low survival rates in patients with colon cancer or lung cancer (114). Several studies have shown that inhibiting integrin or knocking out integrin genes can reduce or reverse drug resistance and the aggressiveness of breast cancer and stomach cancer. Therefore, integrins are considered to be therapeutic targets in a variety of cancers. Abituzumab is an antibody against integrins. In clinical trials, use of abituzumab was associated with increased progression-free survival and response rates in patients with metastatic colorectal cancer and high integrin expression (115). Cilengitide is a selective integrin inhibitor that has been studied in a series of Phase II/III studies in non-small cell lung cancer, pancreatic cancer, and prostate cancer (116–118). However, inhibition of activation of TGF-β has hampered development of abituzumab because of the effects on homeostasis and the risk of serious adverse events. However, Van Aarsen et al. found that TGF-β signaling did not need to be prevented completely, and only the phosphorylated portion of SMAD2/3 was suppressed, and its collagen expression was significantly reduced (119). For example, BG00011, an anti-αvβ6 monoclonal antibody, significantly reduced bronchoalveolar levels of phosphorylated SMAD2 in patients with idiopathic pulmonary fibrosis in a Phase IIA clinical trial, but the trial has been halted owing to safety concerns (120).

### 3.4 Blocking the TGF-β receptor

Blockade of the receptor inhibits the kinase activity of TGF-β, thereby preventing typical and atypical signaling downstream, where TGF-β1 receptors are associated with signal transduction by SMADs. Therefore, these receptors are attractive targets for inhibition. TGF-β receptor inhibitors include vactosertib and galunisertib. Vactosertib was found to inhibit metastasis of breast cancer and enhance anti-tumor T cell immunity and antigen diffusion in mouse models of breast cancer (121). To date, clinical trials of vactosertib have focused on the treatment of cancer, and the efficacy of this agent in human fibrotic diseases is yet to be investigated. Galunisertib is a TβRI kinase inhibitor that is showing promising anticancer activity in breast, colon, lung, and hepatocellular carcinoma xenografts. In a mouse model of colorectal cancer, clinically relevant doses of galunisertib were used to enhance the antitumor activity of anti-programmed death-1 ligand 1 (PD-L1) therapies (anti-mouse PD-L1 clones, which resulted in regression of tumors and enhanced activation of T cells) (122). The aforementioned studies further suggest that, in contrast to other receptor blockers, Galunisterib can remarkably decrease adverse events and toxicity, particularly cardiovascular toxicity.

### 3.5 Blocking intracellular signal transduction

Targeted inhibition of intracellular Smad signaling molecules enables prevention of TGF - β activity triggered by other signaling pathways. This approach differs from blocking TGF - β at the ligand or receptor level. In the classical pathway, interfering with formation of SMAD 2/3 and SMAD 4 complexes reduces the expression of TGF-β response genes, which is a useful way of reducing the risk of negative outcomes (123). Using thioredoxin A as a scaffold, Cui et al. developed three peptide aptamers that can help disrupt subpopulations of TGF-β reactions (124). LY2157299 (Galunisertib) has been shown to inhibit lung cancer and breast cancer cell growth, inhibit the activity of TGF-β receptor I, reduce phosphorylation of SMAD2 and SMAD3, and indirectly affect the formation and function of SMAD2/3/4 complexes, and has been used to evaluate therapeutic effects in a variety of cancer clinical trials (125, 126). SB-431542 is a small-molecule selective inhibitor of ALK-5 that inhibits TGF-β-mediated transcription of renal cancer proteins. However, due to its unstable pharmacokinetics, it has only been studied *in vitro (* 127, 128). Overall, targeting only one signaling pathway is impractical and inefficient in view of the complexity of carcinogenesis. Recent clinical studies have demonstrated the therapeutic potential of TGF-β inhibitors used in combination with adjuvant treatments. TGF-β has a pleiotropic effect on normal physiological function and in tumorigenesis, and thus long-term inhibition of TGF-β and related signaling pathways may produce adverse reactions (129). Therefore, fine-tuning the downstream signaling pathway of TGF-β rather than eliminating it completely at the ligand level would be a better therapeutic strategy. TGF-β signaling mediators, including SMAD2 and SMAD3, vary in their sensitivity to stimulation by TGF-β and bind to different transcription factors, thereby regulating expression of different genes.

## 4 Potential strategies and future prospects for TGF-β inhibitors

As of August 2023, 124 TGF-β blocking agents had been identified worldwide, two of which have received regulatory approval and 73 are still in the clinical research stage. Six agents are in clinical Phase III trials, 33 in clinical Phase II trials, 32 in clinical Phase I trials, and two in clinical application studies (18, 130). These clinical trials are summarized in **Table 1**. As can be seen in this table, inhibition of the TGF-β pathway remains an active area of investigation in cancer research. TGF-β-targeted neutralizing antibodies, vaccines, antisense oligonucleotides, and small molecule inhibitors have been studied in solid tumors in clinical trials (131). Belagenpneumatucel–L is an anti-cancer vaccine developed for non-small cell adenocarcinoma (NSCLC) that theoretically increases the immunogenicity of allogeneic lung cancer vaccine cells, resulting in an immune tumor response (24). However, owing to pleiotropic and safety issues, anti-TGF-β agents are difficult to develop. Fresolimumab appears to be relatively safe in clinical trials, and 10 μM has been identified as the optimal dose for clinical development of this agent. Vincenti et al. reported that inhibition of TGF-β delayed wound healing and caused mild gingival bleeding (132, 133). Adverse events are still higher compared to targeting TGF-β2 mRNA, but this finding could mean that future TGF-β-targeted therapies developed at the protein level will have a better safety profile than those developed at the gene level. Bintrafusp alfa is a bifunctional fusion protein that targets TGF-β and PD-L1 and is formed by fusing the extracellular domain of TGF-βRII with a human IgG1 monoclonal antibody that blocks PD-L1. This agent blocks both the TGF-β and PD-L1 immunosuppressive signaling pathways at the same time. It has been demonstrated that inhibition of both these pathways using a bi-functional approach has better antitumor activity relative to TGF-β “traps” and anti-PD-L1 antibodies. In mouse tumor models, bintrafusp alfa significantly reduced fibrosis, helped reduce local drug resistance, and was more effective in tumor regression on day 24 compared with anti-PD-L1 or anti-TGF-β alone (134). In mouse models of breast and colon cancer, Knudsom et al. demonstrated that bintrafusp alfa not only blocked activation of TGF-β signals in the TME but also significantly reduced the transduced TGF-β signal (135). Preclinical data indicate that bintrafusp alfa reduces the expression of vascular endothelial growth factor (VEGF) in cancer - associated fibrosis and the subsequent angiogenesis by sequestering TGF - β. It may also restore normal vascular homeostasis, thus facilitating drug delivery and the infiltration of T cells into the tumor microenvironment (TME) (134, 136, 137). Simultaneous targeting of two non-redundant immunosuppressive pathways may have superior antitumor activity. Liu et al. conducted a clinical expansion Phase I treatment for advanced solid tumors. They discovered that a dose of 30mg/kg demonstrated the optimal anti - tumor activity. Notably, compared to other doses, there was no significant increase in toxicity. Among the treated patients, 37% showed a reduction in the target - lesion tumor (138, 139).

**Table 1:** Shows the summary of various strategies of TGF-β inhibition in clinical trials.

Strategy	Target	Type	Treatments	Diseases	Clinical trials
**Blocking synthesis**	TGF-β2 mRNA	ASO	Trabedersen	Pancreatic ductal adenocarcinoma and malignant pleural mesothelioma	NCT06079346 (phase 2/3)and NCT05425576 (phase 2)
	TGF-β2 mRNA	ASO	TASO-001	Solid tumors	NCT04862767 (phase1)
**Blocking ligand**	TGF-β1, TGF-β2	Antibody	NIS793	Colorectal cancer, pancreatic cancer and MDS	NCT04952753 (phase2), NCT05417386 (phase1), NCT04390763 (phase2) and NCT04097821 (phase1/2)
	TGF-β2	Antibody	Lerdelimumab	Fibrosis (after surgery)	CAS285985-06-0
	TGF-β1, TGF-β2, TGF-β3	Antibody	Fresolimumab	Breast cancer, lung caner and IPF	NCT01401062 (phase2), NCT02581787 (phase1/2) and NCT00125385 (phase1)
	TGF-β1, TGF-β3	Soluble receptor	AVID200	Solid tumor and myelofibrosis	NCT03834662 (phase1) and NCT03895112 (phase1)
**Blocking latent activation**	Integrin	Antibody	SRK-181	Solid tumor	NCT04291079 (phase1)
	Integrin	Small molecule inhibitor	GSK3008348	IPF	NCT03069989 (phase1)
**Blocking receptor**	ALK5	Small molecule inhibitor	Galunisertib	Prostate cancer and pancreatic cancer	NCT02452008 (phase2) and NCT0234160 (phase1)
	ALK5	Small molecule inhibitor	Vactosertib	Urothelial carcinoma and solid tumor	NCT04064190 (phase2) and NCT02160106 (phase1)
	ALK5	Small molecule inhibitor	LY3200882	Solid tumor	NCT02937272 (phase1)
**Blocking intracellular signaling**	JNK	Small molecule inhibitor	Tanzisertib	IPF	NCT01203943 (phase2)

The bold values correspond to the five strategies of TGF-β inhibition in clinical trials: Blocking synthesis; Blocking ligand; Blocking latent activation; Blocking receptor; Blocking intracellular signaling.

Many TGF-β-targeted therapies are presently under investigation for their effects when used in combination with anti-PD-L1 therapies, particularly in cancers that do not respond well to PD-L1 monotherapy. Gemogenovatucel-T is being used alone or in combination with atezolizumab (NCT03073525) or duvaliumab (NCT02725489) in advanced gynecological cancers (140). Valeria et al. found that the neutralizing antibody 1D11 combined with anti-PD-1 helps T cells penetrate into the center of a tumor and enhances anti-tumor immunity (141, 142). Simultaneous administration of anti-TGF-β, anti-VEGF (Y332D), and anti-PD-1 inhibited tumor growth and metastasis in lung metastasis models and was superior to anti-VEGF and anti-TGF-β alone in reducing nodules in lung tissue (143, 144). Furthermore, anti-PD-1 and Y332D significantly increased survival time in mouse models of hepatocellular carcinoma (145). Y332D promotes transition of tumors from “cold” to “hot”, potentially increasing the sensitivity of PD-1 antibodies. Studies in pancreatic cancer cell lines and xenografts in mice have found that combination therapy consisting of galunisertib, a TGF-β receptor inhibitor, and lapatinib, an inhibitor of both the epidermal growth factor receptor and human epidermal growth factor receptor 2, reduces tumor growth and metastasis by inhibiting lymphangiogenesis and angiogenesis (147). Other studies in cultured cell lines and mouse xenografts have shown that LY2109761, an inhibitor of TGFBR1 and TGFBR2 kinase activity, has antitumor effects that are synergistic with those of gemcitabine and reduces metastasis (148, 149). Galunisertib in combination with gemcitabine extends survival of unresectable pancreatic cancer in humans (150). In conclusion, these results offer evidence suggesting that combination therapy involving TGF-β inhibitors may present a more favorable safety profile and be more easily managed compared to TGF-β monotherapy. Traditional Chinese Medicine is a multi-targeted treatment, in which each target can cooperate with others with a low risk of adverse effects. Therefore, there are many possibilities for integration of traditional Chinese and Western medicine. Dachaihu decoction reduced expression of TGF-β in a rodent model of non-alcoholic fatty liver disease, and psoralen decreased TGF-β levels in bleomycin-induced pulmonary fibrosis (146, 151). No biomarker of an inhibitory response to TGF-β has been identified to date. More studies of immunophenotypes and the characteristics of TGFβ-related gene expression, as well as genomic biomarkers, are warranted.

TGF-β is a ubiquitous, multifunctional cytokine that is believed to be a central pathway in the development and progression of cancer. TGF-β inhibition strategies have demonstrated beneficial effects in mouse models of cancer. Considering the pleiotropic nature of TGF-β and its role in biological homeostasis, the safety of TGF-β antagonists in human patients must be carefully evaluated. Considering that targeting TGF-β signaling by inhibition of its receptors or neutralizing all TGF-β subtypes may be associated with serious adverse events, such as keratoacanthoma, squamous cell carcinoma, and impaired immunity. Many of these approaches have shown promising anti-metastasis effects in preclinical models and potential for further clinical development.

## 5 Limitations of anti–TGF-β therapy

Preclinical and clinical data of anti-TGF-β therapies suggest promise for targeting this pathway as anti-cancer therapies. However, anti-TGF-β therapy has a number of limitations:

At present, most of the data for anti-TGF-β therapy are derived from animal experiments, which are difficult to fully simulate the complexity and heterogeneity of human tumors. However, some reports used in human experiments have pointed to adverse effects in some patients. For example, in a multicenter, phase II clinical trial, researchers used fresolimumab, an anti-TGF-β monoclonal antibody, in patients with advanced pancreatic cancer in an attempt to block the TGF-β signaling pathway to curb tumor progression, but the overall survival rate of patients was not significantly improved (152, 153). In addition, some early clinical studies in non-small cell lung cancer (NSCLC) have introduced anti-TGF-β therapies in combination with conventional chemotherapy, and some patients have not only not benefited from the combination therapy, but their disease has accelerated their deterioration (154–157). This has the opposite effect of TGF-β in the stage of tumorigenesis, so it is difficult for anti-TGF-β to accurately distinguish the stage of the tumor, which may counteract the original antitumor effect and accelerate the deterioration of the tumor.

For the treatment of tumors, a combination of drugs is often used, such as TGF-β is combined with radiotherapy, chemotherapy, immunotherapy and other drugs (90, 158). However, the TGF-β signaling pathway intersects and overlaps with many other anticancer drug pathways, and when combined, complex interactions between drugs may cancel out each other’s effects or cause unpredictable toxicity stacking. For example, the combination of anti-TGF-β drugs with platinum-based chemotherapy drugs may alter the uptake and metabolism of chemotherapy by tumor cells, making chemotherapy response unstable (159, 160). The sequence and time interval of combination therapy and the optimal timing of intervention in different tumor stages are different, and if anti-TGF-β therapy is intervened too early or too late, it will not be able to form a synergistic effect with other therapies.

In addition to the antitumor effects of TGF-β, this cytokine is also important for normal tissues homeostasis. Since TGF-β is involved in many normal physiological processes, systemic inhibition of TGF-β may have harmful side effects, and when anti-TGF-β drugs are applied systemically, healthy tissues will also be affected, such as interfering with the normal renewal of liver and kidney cells, resulting in abnormal liver and kidney function, and may also affect the repair of the gastrointestinal mucosa, causing digestive disorders (90, 161, 162).

## 6 Conclusion

**Figure 2:**
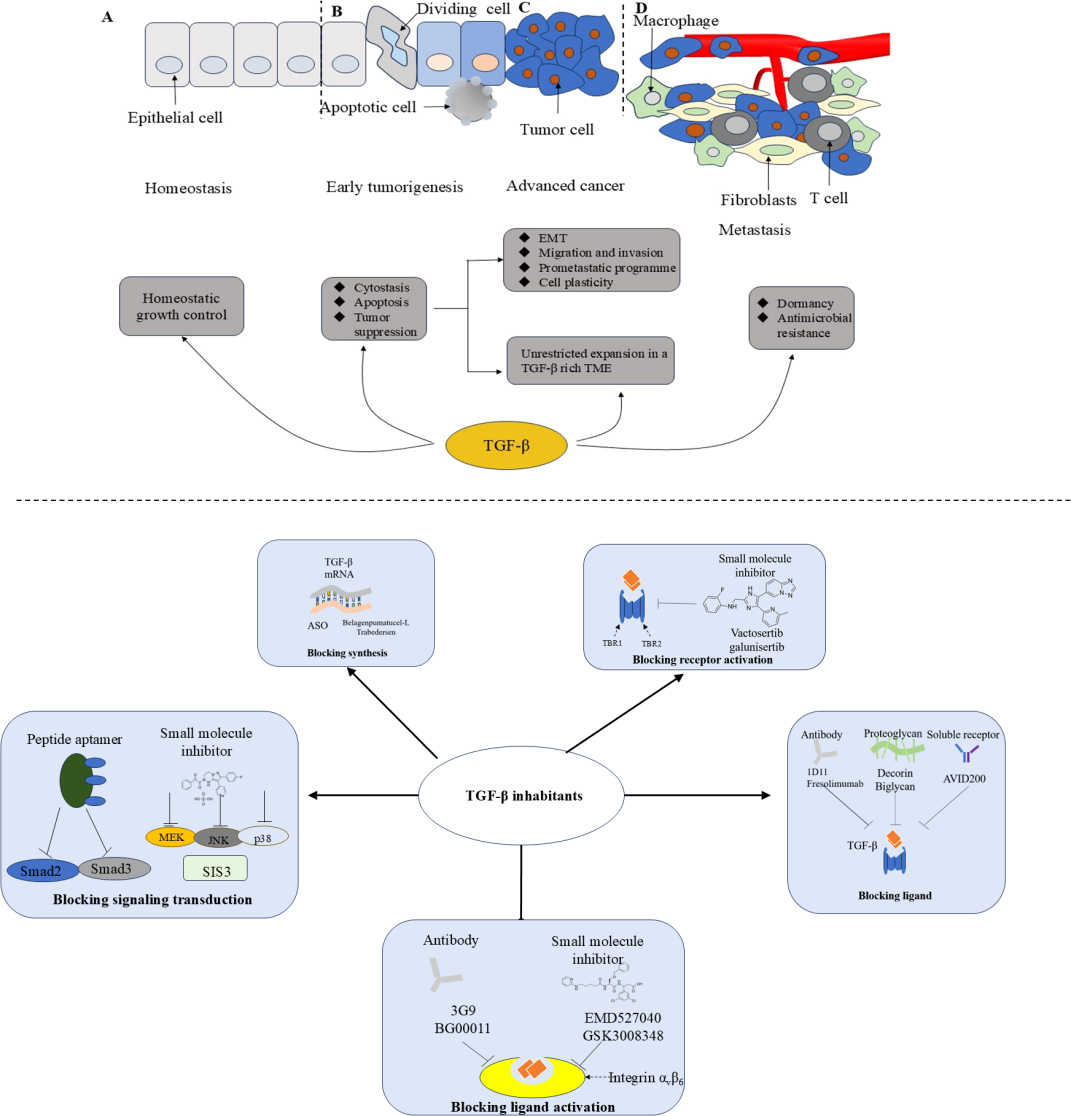
The function of TGF-β during tumor progression and therapeutic strategies of TGF-β inhibition. ASO, antisense oligonucleotides; EMT, Epithelial-mesenchymal transition; TME, Tumor micro-environment. During tumorigenesis, TGF - β transitions from acting as a tumor suppressor in the premalignant stages to promoting tumor growth in the later stages of the disease, a process that ultimately leads to metastasis. **(A)** Normal epithelium; **(B)** Early tumorigenesis; **(C)** Advanced cancer; **(D)** Invasive metastatic cancer.

The TGF-β signaling pathway is essential under normal physiological conditions but is also involved in progression of cancer, and thus has attracted considerable research interest in recent years. TGF-β shapes the TME in a way that is conducive to tumor progression (**Figure 2**). Therefore, blocking TGF-β has the potential to reduce invasion and migration of tumor cells and holds promise for broad application in clinical practice. However, the potential adverse effects of TGF-β inhibitors have hindered their clinical application. The results of recent clinical trials have raised concerns regarding the toxicity of TGF-β inhibitors and suggest the possibility that inhibiting the TGF-β signaling pathway may worsen rather than improve the outlook for patients with cancer. At present, none of these agents has been approved for the treatment of cancer or fibrosis. Inflammation and bleeding are common adverse effects of TGF-β therapy, and TGF-βR inhibitors have been found to cause significant cardiotoxicity. Therefore, there is a need for more targeted clinical treatment. Targeted nanotechnology-based interventions have been used as an available measure to improve treatment. Considering the pleiotropic effect of TGF-β, targeting its downstream signal transduction may identify better targets for cancer therapy. Finally, patients need to be carefully selected for participation in clinical trials, and the indications and dosages of drugs should be carefully defined to limit both targeted and off-target adverse effects.
